# The association of self-regulation with weight loss maintenance after an intensive combined lifestyle intervention for children and adolescents with severe obesity

**DOI:** 10.1186/s40608-016-0140-2

**Published:** 2017-04-25

**Authors:** Jutka Halberstadt, Emely de Vet, Chantal Nederkoorn, Anita Jansen, Ottelien H. van Weelden, Iris Eekhout, Martijn W. Heymans, Jacob C. Seidell

**Affiliations:** 1 Department of Health Sciences and the EMGO Institute for Health and Care Research, VU University Amsterdam, Amsterdam, The Netherlands; 2 Sub-department Communication, Philosophy and Technology: Centre for Integrative Development, Chairgroup Strategic Communication, Wageningen University, Wageningen, The Netherlands; 3 Faculty of Psychology and Neuroscience, Department of Clinical Psychological Science, Maastricht University, Maastricht, The Netherlands; 4 Department of Epidemiology & Biostatistics and the EMGO Institute for Health and Care Research, VU University Medical Center, Amsterdam, The Netherlands

**Keywords:** Childhood obesity, Weight loss maintenance, Self-regulation, Inhibition, Reward

## Abstract

**Background:**

Knowledge is limited on the role the ability to self-regulate plays in the long-term outcome of obesity treatment in children and adolescents with severe obesity. The purpose of this study was to determine whether the ability to self-regulate after an one year intensive, partly inpatient, combined lifestyle intervention is associated with weight loss maintenance in children and adolescents with severe obesity.

**Methods:**

One hundred twenty participants (8–19 years) with an average SDS-BMI of 3.41 and their parents/caregivers were included in an intervention study. As primary determinant of weight loss maintenance, general self-regulation ability was evaluated using two behavioral computer tasks assessing inhibitory control and sensitivity to reward.

**Results:**

There was no association between inhibitory control at T12 and ∆SDS-BMI between T12 and T24 (β = 0.0002; CI 95% = −0.0010–0.0014; *P* = 0.761). There was also no relation between sensitivity to reward at T12 and ∆SDS-BMI between T12 and T24 (β = −0.0028; CI 95% = −0.0075–0.0019; *P* = 0.244). None of the psychosocial factors that were examined as moderators, showed a statistically significant interaction, except for parental feeding style (*P* = 0.023).

**Conclusions:**

The ability to self-regulate after an intensive, partly inpatient, multidisciplinary one year intervention for severe obesity in children and adolescents was not associated with the ability to maintain the achieved weight loss during the following year. Factors that explain the large range of long term outcomes need to be elucidated.

**Trial registration:**

Netherlands Trial Register (NTR1678, registered 20-Feb-2009).

## Background

The long-term results of combined lifestyle interventions for children and adolescents with obesity are generally disappointing, mainly because of a lack of weight loss maintenance [1]. However, treatment success varies substantially between persons. It is not clear what factors determine these differences in treatment outcome [2]. It has been proposed that differences in self-regulation abilities may explain differences in long-term weight loss [3–11]. In this study we investigate this hypothesis and in addition explore whether other psychosocial factors may modify this relation.

Self-regulation can be defined as any effort, conscious and non-conscious, by individuals to alter their thoughts, emotions, attention, impulses and behavior [12] in order to attain and maintain personal goals [13]. When controlling food intake, two facets of self-regulation seem critical: inhibitory control and sensitivity to reward [14–16]. Inhibitory control is the capacity to inhibit impulses and responses [16]. Sensitivity to reward encompasses both the sensory pleasure the reward produces and the degree of motivation to get hold of the reward [16].

Research with both adults and children indicated that a lower general ability to self-regulate was associated with obesity [4, 5, 14, 17]. More specifically, obese children and adolescents generally show a higher sensitivity to reward and a lower inhibitory control compared to lean children and adolescents [6, 14, 17–24]. Furthermore, an impaired ability to self-regulate is predictive for drop-out of weight management programs, less weight loss during weight loss interventions and a lower weight loss maintenance [5, 7, 14, 25]. Nevertheless, research indicates that the ability to self-regulate eating behavior can be improved through behavioral treatment [4, 8].

In sum, a low ability to self-regulate may predict less treatment success and should be a target of the intervention when treating children and adolescents for their obesity. This is especially important for the weight loss maintenance phase. Simply stated: after treatment ends, it requires self-regulation to continue having a lower calorie intake and a higher energy expenditure. Therefore, getting insights in the ability to self-regulate can provide useful information on what determines long-term weight loss. To what extent the ability to self-regulate is related to the outcome of behavioral interventions in children and adolescents with severe obesity is not yet known.

The main objective of this study was to determine whether the ability to self-regulate is associated with long-term weight loss in children and adolescents with severe obesity that participate in an intensive, partly inpatient, combined lifestyle intervention. With a special focus on testing the main hypothesis that the ability to self-regulate after the intervention is associated with the ability to maintain the achieved weight loss during the following year, regardless of the ability to self-regulate at baseline. An additional objective was to identify in an explorative analysis other psychosocial factors, related to competence, motivation, relatedness and outcome expectations, that may modify the relation between the general ability to self-regulate and weight loss maintenance. The rationale, design and methods of this study have been described in detail elsewhere [26].

## Methods

### Study design

The study was designed as an intervention study of children and adolescents with severe obesity that underwent an intensive, partly inpatient, combined lifestyle intervention during one year. Measurements were performed at three time points: at baseline (start of treatment; T0), at the end of treatment (12 months after baseline; T12) and at the end of follow-up (24 months after baseline; T24). More details on the measurements can be found elsewhere [26].

The study was an expansion of a study that had the objective to determine the effectiveness and cost-effectiveness of the treatment program [27]. The Medical Ethics Committee of VU University Medical Center Amsterdam approved the study design, protocols and informed consents procedure. The trial was registered in the Netherlands Trial Register (NTR1678, registered 20-Feb-2009).

### Setting

Data were collected between August 2009 and July 2013 at the childhood obesity clinic Heideheuvel (part of Merem Treatment Centers) in Hilversum, the Netherlands. Heideheuvel is the only clinic in the Netherlands that offers inpatient care for childhood obesity.

### Study population

The aim was to include 120 participants (40 children and 80 adolescents). This number was partly based on the calculated needed sample size of 40 children and 40 adolescents for the part of the study that was a randomized controlled trial and that had the objective to determine the effectiveness and cost-effectiveness of the treatment program [27]. Due to recruitment difficulties for the children, 10 extra adolescents instead of children were included. Due to extra capacity at the treatment center in the same time period, it was possible to include another 40 adolescents for the current study that was not a randomized controlled trial. The total of 120 participants with severe obesity and their parents/caregivers included in this study were therefore: 30 children (8–13 years) and 90 adolescents (13–19 years). For the randomized controlled trial the intervention had either a two months or a six months inpatient period. The aim was to include 20 children and 20 adolescents in both variations of the intervention. The extra treatment that was available for 40 patients in all cases had a two months intervention period due to the organizational capacity of the treatment center. Because there was a waiting list for adolescents and not for children, the extra 40 patients were all adolescents. In total eighty participants (20 children and 60 adolescents) underwent a two months inpatient period and 40 (10 children and 30 adolescents) a six months inpatient period. The criteria for inclusion and exclusion are described elsewhere [26]. Three participants dropped out of the treatment around the start of the study and were replaced by participants from the waiting list. The latter were used in the analyses.

The chosen division between children (up to age 13) and adolescents (from age 13 up to age 19) is based on the Dutch school system where children finish elementary school around age 12 and by the time they are 13 they are all enrolled in some form of secondary education and have started a different phase of their lives. To acknowledge this phase change, the treatment program offers different treatment groups for these different age groups.

If more than one of the parents/caregivers was available at baseline, they were asked to determine which of them was the most involved care taker for the child or adolescent included in the study. That parent/caregiver was asked to participate in the study at the different measuring points.

Both the participants (from age 12) and their parents/caregivers provided written informed consents. A 20 Euro gift voucher was offered to reimburse travel expenses for the study participants.

### Intervention

The patients were treated in sequential groups of 10 children or 10 adolescents. The treatment program comprised an intensive combined lifestyle intervention by a multidisciplinary team. This team consisted of psychologists, social workers, group coaches, pediatricians, dieticians, nurses, physiotherapists and exercise therapists. The program, that lasted one year, had a period of inpatient treatment during weekdays of either 2 months and biweekly return visits of 2 days during the next 4 months or 6 months, followed by 6 monthly return visits of 2 days. The intervention required active and frequent participation of the parents/caregivers. The emphasis of the intervention was on nutrition, exercise and behavior, in line with national and international guidelines [28–30].

Behavior change techniques were used to improve the general ability to self-regulate. The intervention further addressed themes like disordered eating behavior, self-worth, self-efficacy, behavioral and emotional problems, autonomous motivation, body image, outcome expectations, mood disorders, eating and exercise behavior, interaction between parent and child, parental feeding styles, relationships with peers, body acceptance and coping.

More information on the contents of the treatment and its components can be found elsewhere [26, 27].

### Measurements

#### Outcome measure - SDS-BMI change

Height and weight were measured at the childhood obesity clinic. Height was recorded with a Holtain stadiometer with an accuracy of 1 mm that was fixed on the wall. The stadiometer was calibrated before every first measurement. Height was recorded three times of which the average was calculated. Weight was measured in light clothing without shoes and recorded with a calibrated SECA digital weight chair that had a limit of 230 kg and an accuracy of 0.005 kg. Height and weight were used to calculate BMI and SDS-BMI (Standard Deviation Score of Body Mass Index) with the Growth Analyzer (www.growthanalyser.org; version 3.5, computer program by “Stichting Kind en Groei”, downloaded in July 2010) using the fourth Dutch nationwide growth study of 1997 as reference since this was the most recent national growth study at the time the study started in 2008 and the first results were published. International cut-points for severe obesity were not available at the time of the recruitment. Weight change was calculated with the gender and age-specific SDS-BMI, that indicates how many standard deviations a measurement is above or below the median of the distribution.

The primary outcome measurement of this study was the SDS-BMI change between the end of treatment (T12) and the end of follow up (T24), indicating the weight loss maintenance. In addition we assessed the SDS-BMI change between baseline and the end of treatment (initial weight loss) and between baseline and the end of follow-up (total weight loss).

#### Primary determinant - Self-regulating ability

As primary determinant of (sustained) weight loss, the general ability to self-regulate was assessed in the children and adolescents with two computerized behavioral tasks: the Stop Signal Task [31] that assesses inhibitory control and the Balloon Analogue Risk Task [32] that assesses sensitivity to reward.

The main outcome of the Stop Signal Task is the Stop Signal Reaction Time (SSRT), measured in milliseconds (ms). A longer SSRT means that the participant has more difficulties to inhibit his/her response and indicates less inhibitory control.

The main outcome of the Balloon Analogue Risk Task is the Adjusted Value (AV), indicating the average number of pumps excluding balloons that exploded. A higher AV points to a higher sensitivity to reward, which indicates a lower ability to self-regulate.

The computer tasks are described in more detail elsewhere [26].

#### Potential moderators and confounders

Several psychosocial factors were explored as potential moderators of the relation between the ability to self-regulate and weight loss maintenance with questionnaires for the participants and their parent/caregiver, as described in detail elsewhere [26]. The following factors were assessed:

General self-efficacy was measured with the General self-efficacy scale (GSE), which assesses one’s sense of personal competence to cope across a variety of demanding or novel situations [33].

Self-worth was measured with the global self-worth scale of the Self-Perception Profile for Children (SPPC) and Adolescents (SPPA) [34–37].

Autonomous Motivation was measured with the Relative Autonomy Index of the Treatment Self-Regulation Questionnaire (TSRQ) that assesses reasons to enter a weight-loss program [38].

Interaction between child and parent was measured with the Parent–child Interaction Questionnaire – Revised (PACHIQ-R) [39] that assesses how the parent evaluates the relationship with the child.

Social competences and social problems as assessed by the parent, were measured with two scales of the Child Behavior Checklist (CBCL/6-18) [40, 41] that assesses competences and behavioral and emotional problems: 1) the social scale and 2) the social problems scale.

Parental feeding style was measured with the Parental Feeding Style Questionnaire (PFSQ) [42], which asks parents to report the frequency with which they use a number of feeding strategies [43].

The affect of the parents was measured with the Positive and Negative Affect Schedule (PANAS) [44, 45], in which parents are asked to indicate how they currently feel emotionally.

Perception of peer body size was measured by asking children and adolescents to select from a range of nine line drawings of silhouettes ranging in body size from very thin to severely obese, the silhouette that most closely resembled how their five best same sex friends look [46–48].

The same silhouette drawings were used to assess outcome expectations. Participants were asked to select the silhouette that most closely resembled how they expected to look after the program.

As other potential moderators, a number of factors were identified: SDS-BMI at baseline, initial weight loss, gender, age at baseline, ethnicity, educational level of the parents and intensity of treatment (inpatient period of 2 or 6 months).

### Statistical analyses

Statistical analyses were carried out using SPSS 20.0 and R statistical software [49]. The analyses were restricted to the participants with complete data, except for the analyses on the baseline characteristics (Table 1) that were also performed for the participants without complete data. Participants were considered to have complete data when they had observed scores for inhibitory control and sensitivity to reward at T12 and an available SDS-BMI score at T12 and T24.

For the participants with complete data, the missing data on the questionnaires were handled by multiple imputation at the item level, in order to have the highest power [50]. When participants missed an entire scale, the scale score was imputed at the scale score level. Multiple imputation was performed using all available data in R statistical software [49] by predictive mean matching using the Multivariate Imputation by Chained Equations (MICE) method [51].

As a sensitivity analysis, all analyses were repeated without the children (*n* = 3, of which 2 in the group with complete data) with an unexpectedly high subscore on the Stop Signal Task measuring inhibitory control (a subscore of ≥61 on the Nstop; the rest of the subscores on the Nstop was ≤50 at all measuring points for all participants) and also without the child (*n* = 1, in the group with complete data) that underwent bariatric surgery before T24, to check whether this would affect the results.

#### Baseline characteristics

For all participants the baseline characteristics were determined. An independent-samples *t*-test was then done to test the differences between the participants with and without complete data on BMI, SDS-BMI, age, inhibitory control and sensitivity to reward at T0.

#### Outcome measure and primary determinant

The means and standard deviations of SDS-BMI at T0, T12 and T24 were determined for the group with complete data and boxplots were made of the SDS-BMI at the three measuring points.

To test the main hypothesis of this study, we looked at inhibitory control and sensitivity to reward after one year of treatment as determinants of weight loss maintenance with the outcome measurement as continuous variable with linear regression analysis. In addition, we assessed the association between the ability to self-regulate at baseline and initial weight loss and total weight loss.

Furthermore, the means and standard deviations of the scores on inhibitory control and sensitivity to reward at T0 and T12 were determined. The change over time of these scores was tested with paired sample t-tests. In addition the intra-class correlation between T0 and T12 was calculated for the scores on both inhibitory control and sensitivity to reward to assess the stability of the scores for the individual participants.

#### Moderators and confounders

Additional explorative analyses were done to examine if the psychosocial characteristics of the participants were moderators of the relation between inhibitory control and sensitivity to reward at T12 and weight loss maintenance in the linear regression models. For this, the cross product of inhibitory control at T12 or sensitivity to reward at T12 with the potential moderators (the psychological factors as measured by the questionnaires) were introduced as interaction terms in the models.

The moderating factors assessed at T12 were used, and the moderators that were only assessed at T0 were accordingly included in the models. Where applicable total scores on the questionnaires, subscale scores, or both were used. Because the scoring on the Self-Perception Profile is different for children and adolescents, the participants were dichotomized according to the median of the T0 scores calculated with age and gender specific scores, to acquire comparable scores. For the other questionnaires, means and standard deviations were calculated.

Furthermore, the described other factors (i.e. SDS-BMI at baseline, initial weight loss, gender, age at baseline, ethnicity, educational level of the parents and intensity of treatment) were assessed, by stratification, as potential moderators of the relation between inhibitory control and sensitivity to reward at T12 and weight loss maintenance.

We also assessed if the psychosocial characteristics and the other factors were confounders.

## Results

### Baseline characteristics

Table 1 presents baseline characteristics of all study participants and of the participants with (*n* = 74) and without (*n* = 46) complete data separately. There were no statistically significant differences between the groups with and without complete data as assessed for the variables BMI, SDS-BMI, age, the scores on inhibitory control and sensitivity to reward and the percentage of boys and girls.

**Table 1:** Participants with (*n =* 74) and without (*n =* 46) complete data compared on several variables at baseline (T0)

	Total	Complete data	Incomplete data
N	120	74	46
BMI	40.2 (6.1)	40.8 (5.5)	39.2 (6.8)
SDS-BMI	3.41 (0.38)	3.44 (0.36)	3.36 (0.40)
Age (y)	14.8 (2.4)	14.9 (2.3)	14.7 (2.4)
Male/Female (%)	32.5/ 67.5	29.7/ 70.3	37.0/ 63.0
Ethnicity (%)			
Native Dutch	60.7	62.0	58.7
Other Western	4.3	4.2	4.3
Non-Western	35.0	33.8	37.0
Level of education parents (%)			
Low	36.7	37.8	34.8
Medium	39.2	40.5	37.0
High	19.2	18.9	19.6
Unknown	5.0	2.7	8.7
Inhibitory control			
SSRT (Stop Signal Task)^a^	261.9 (82.8)	254.7 (87.3)	272.9 (74.9)
Sensitivity to reward			
AV (Balloon Analogue Risk Task)^a^	24.3 (10.9)	24.9 (11.1)	23.5 (10.5)

Data are mean (SD), *SD* standard deviation, *BMI* body mass index, *SDS-BMI* standard deviation score of body mass index, *SSRT* stop signal reaction time measured in ms, *AV* adjusted value defined as the average number of pumps excluding balloons that exploded

^a^ In the group with complete data, at T0 scores on the SSRT were not available for three participants and scores on the AV were not available for two of them

### SDS-BMI change

The SDS-BMI of the participants with complete data on average was 3.44 (0.36) at baseline, was 3.03 (0.63) after one year of treatment (*P* < 0.001) and 3.18 (0.67) at the end of follow up after two years (*P* < 0.001). So on average the participants lost 0.41 SDS-BMI points during treatment, they partly relapsed in the year after treatment and their total weight loss at the end of follow up after two years was 0.26 SDS-BMI points.

**Fig. 1:**
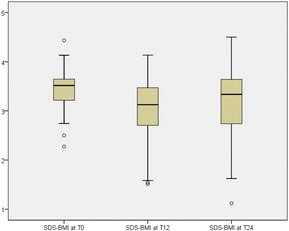
Boxplots of SDS-BMI of participants with complete data (*n =* 74) at baseline (T0), after one year of treatment (T12) and at the end of follow up (T24). Note: The lines in the middle of the boxes indicate the median, meaning 50% of the participants score above this value and 50% below. The borders of the boxes are the 25th (bottom line) and 75th (top line) percentile points, so the boxes contain 50% of the participants at each measuring point. The whiskers, the narrow horizontal lines below and above the boxes, indicate the lowest and highest values excluding the outliers. These outliers, indicated with the small circles, are values that deviate more than one and a half times the height of the box from the top border or the bottom border of the box

Figure 1 shows the large variation of SDS-BMI between participants at all measuring points, especially at T12 and T24.

### Self-regulation ability as determinant of weight loss maintenance

**Fig. 2:**
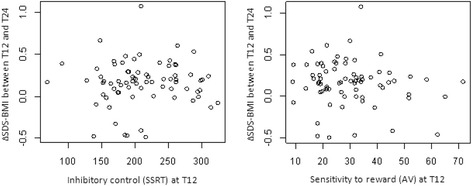
Inhibitory control and sensitivity to reward after one year of treatment (T12) as determinants of weight loss maintenance for participants with complete data (*n =* 74)

Figure 2 shows the results for the main hypothesis: the scores on inhibitory control and sensitivity to reward after one year of treatment (x-axes) in relation to weight loss maintenance at the end of follow up, measured by the change in SDS-BMI between T12 and T24 (y-axes).

The left picture shows the results on the Stop Signal Task. A lower SSRT score indicates a better inhibitory control. The right picture shows the results on the Balloon Task. A lower AV score indicates a lower sensitivity to reward.

There was no association between inhibitory control at T12 and ∆SDS-BMI between T12 and T24 (β = 0.0002; CI 95% = −0.0010–0.0014; *P* = 0.761). There was also no association between sensitivity to reward at T12 and ∆SDS-BMI between T12 and T24 (β = −0.0028; CI 95% = −0.0075–0.0019; *P* = 0.244).

We also looked at inhibitory control and sensitivity to reward at baseline (T0) as determinants of initial weight loss and total weight loss. None of these showed statistically significant associations either.

### Change in self-regulation ability

Table 2 shows that the scores on inhibitory control decreased between T0 and T12, indicating an improvement of the inhibitory control and a better ability to self-regulate. On average the participants statistically significantly improved their inhibitory control.

Table 2 also shows that the scores on sensitivity to reward increased between T0 and T12, indicating a higher sensitivity to reward and a deteriorated ability to self-regulate. The scores on sensitivity to reward on average deteriorated statistically significantly.

**Table 2:** Inhibitory control and sensitivity to reward of the participants with complete data (*n =* 74) at baseline (T0) and after one year of treatment (T12) and their intra-class correlation

	T0	T12	P-value*	Intra-class correlation
Inhibitory control				
SSRT (Stop Signal Task)^a^	254.7 (87.3)	212.5 (54.6)	<0.001	0.41
Sensitivity to reward				
AV (Balloon Analogue Risk Task)^a^	24.9 (11.1)	30.0 (13.1)	<0.002	0.44

Data are mean (SD), *SD* standard deviation, *SSRT* stop signal reaction time, SSRT is measured in ms, *AV* adjusted value defined as the average number of pumps excluding balloons that exploded

*Paired sample *t*-test

^a^ In the group with complete data, at T0 scores on the SSRT were not available for three participants and scores on the AV were not available for two of them

### Potential moderators

Table 3 describes the psychosocial characteristics of the participants at T12.

None of the psychosocial factors that were examined as moderators of the relation between inhibitory control and sensitivity to reward at T12 and weight loss maintenance, showed a statistically significant interaction, except for the ‘control over eating’ subscale of the Parental Feeding Style Questionnaire at T12 (β = −0.0022; CI 95% = −0.0041–-0.0004; *P* = 0.023). With a high score on this subscale and a high score on the SSRT (indicating a low inhibitory control and a low ability to self-regulate) at T12, more weight maintenance was achieved. With a low score on the subscale ‘control over eating’ and a high score on the SSRT, the reversed effect of less weight maintenance was achieved, implying there is effect modification.

**Table 3:** Description of psychosocial characteristics of children and adolescents (*n =* 74) after one year of treatment (T12)

	T12
General self-efficacy	3.05 (0.62)
Self-worth	46.2^a^
Autonomous motivation	2.51 (1.37)^b^
Interaction between child and parent evaluated by parent	
Conflict resolution	45.3 (6.0)^b^
Acceptance	35.1 (4.2)^b^
Social competences	7.22 (2.12)
Social problems	2.80 (2.93)
Parental feeding style	
Emotional feeding	1.42 (0.62)
Instrumental feeding	1.32 (0.53)
Prompting/encouragement to eat	3.11 (0.79)
Control over eating	3.38 (0.67)
Affect of the parent	12.3 (11.5)^b^
Perception of peer body size	3.74 (0.85)
Outcome expectations	1.94 (1.07)^b^

Data are mean (SD), *SD* standard deviation

^a^ Percentage of children and adolescents with high self-worth

^b^ Data collected at T0

None of the other factors assessed as potential moderators (i.e. SDS-BMI at baseline, initial weight loss, gender, age at baseline, ethnicity, educational level of the parents and intensity of treatment) affected the relation between inhibitory control and sensitivity to reward at T12 and weight loss maintenance. These factors also did not modify the relation between inhibitory control and sensitivity to reward at T0 and initial weight loss and total weight loss.

All analyses were repeated for the sample without the participants with the unexpectedly high score on the Stop Signal Task and for the sample without the participant that underwent bariatric surgery between T12 and T24. This did not make a statistically significant difference on the outcomes for any of the variables.

All analyses were also repeated with the not imputed data which did not affect the outcomes.

## Discussion

### Main findings

The results of this study showed no statistically significant association between the ability to self-regulate after one year of treatment and subsequent weight loss maintenance.

Moreover, no statistically significant association was observed for the ability to self-regulate before treatment and initial weight loss or total weight loss.

The association between the ability to self-regulate and weight loss maintenance was also not observed after adjustment for potential moderators or confounders, except for parental control over eating behavior. This is the only statistically significant observation in a large number of tests and therefore may be a chance finding.

The main finding of this study is in contrast to some other studies, in which a better self-regulation was found to be correlated with more initial weight loss or more total weight loss. For instance, a study of obese and severely obese adolescents attending a residential camp offering a multidisciplinary program for obesity, showed adolescents having a better inhibitory control at baseline attained more initial weight loss as assessed at the end of treatment [52]. Another study of obese and severely obese adolescents, attending a one-year multidisciplinary residential treatment for obesity, showed adolescents having a better inhibitory control attained more initial weight loss [14]. A third study of obese and severely obese adolescents, attending a 5 or 6 week inpatient multidisciplinary treatment for obesity, showed adolescents having less inattention and hyperactivity/impulsivity at baseline, attained more total weight loss as assessed at the end of follow up one year after the end of treatment (but not more initial weight loss as assessed at the end of treatment) [53]. The results of this last study indicate that self-regulation is especially important after the intensive treatment phase and the external regulation this encompasses, has ended.

The contrasting results of these other studies might be caused by differences in the duration, intensity or contents of the applied interventions that took place in various countries. Also, the content of the interventions is often not described in detail and therefore not exactly known. Moreover, the patient groups in these different studies may not be comparable due to differences in, for example, the treatment history or presence of comorbidities. The current study included participants with severe obesity who suffered from various obesity related morbidities [54]. As was indicated by the health care professionals involved in the treatment, they often came from families with complex social and health problems. Also, before their referral to the clinic they were diagnosed by their referring pediatrician as not having been able to sufficiently respond to outpatient treatments. This may imply that the included participants had substantial difficulties in losing weight and a high likelihood to relapse. Although the participants varied considerably in the scores on the computer tasks, these characteristics indicate that, in general, they had poor self-regulating skills in maintaining dietary habits. These various characteristics and circumstances make it plausible that the varying outcomes of the different treatments might be related to any of these factors. Still, self-regulation itself also remains a plausible factor in long term weight loss maintenance although findings are inconsistent [55, 56].

### Strengths

To our knowledge this is the first study to prospectively assess the role of inhibitory control and sensitivity to reward in weight loss maintenance in a large sample of children and adolescents with severe obesity undergoing an intensive combined lifestyle intervention.

This study focused on a growing but often disregarded patient group. With an average SDS-BMI of 3.41 at baseline, they represent the extremes of the obesity spectrum in children and adolescents. The number of participants, 120 children and adolescents and their parents/caregivers, is relatively high compared to other intervention studies in a similar population. Further, the drop out was quite low with 82.5% (99 of the 120) of the participants completing the treatment and 75.8% (91 of the 120) of the participants remaining in the study at the end of follow up. Although complete data were available for only 61.7% (74 of the 120) of the initial participants, their baseline characteristics were not statistically different from the rest of the participants. This implies that there was no selective drop out from the study. With an intervention of one year and a follow up of two years after baseline, the duration of this study was relatively long compared to other studies. This strength made it possible to not only assess initial weight loss, but also weight loss maintenance.

### Limitations

In addition to these strengths, some limitations need to be acknowledged. The computer tasks, that were used because they provide a relatively objective measurement method, might not have captured all relevant aspects of the concept of self-regulation ability. Furthermore, it is difficult to interpret the absolute scores on the computer tasks used in this study. Validated norms do not exist and comparisons to other studies must be done with caution because of the use of other versions of the computer tasks, use of different kinds of computers and different characteristics of the study populations [5, 14, 52, 57–59].

Another issue that needs to be addressed is the use of SDS-BMI as main outcome measure. SDS-BMI was the best measure available and practically applicable to capture change in body fatness. But neither SDS-BMI or BMI are ideal outcome measures. The pubertal stage influences body composition and fat distribution partly independent of these measures which makes them less reliable [60].

### Research recommendations

The finding that the amount of control of the parents over eating behavior was a moderator of the relationship between self-regulation at the end of treatment and weight loss maintenance, might be an effect of interest. More research on the relationship between parenting and self-regulation in the context of weight control in children, is needed.

In addition, it is important to acknowledge that although this study did not find self-regulation as measured with behavioral computer tasks to be associated with weight loss maintenance and no moderators of this effect were found, there must exist psychosocial, physical, environmental, socio-economic and/or cultural factors that explain the individual differences in treatment success. Looking for those factors is an important task that can further the knowledge on this specific population and thereby improve the available treatment options.

### Implications for clinical practice

Irrespective of the possible causes, the fact that on average the treated patients were only partly able to maintain their weight loss in the year after the intervention ended, indicates there is a need for a phase of relapse prevention as part of the offered care after an intensive combined lifestyle program like the one described. This is in line with national [28, 29] and international guidelines [30] for the treatment of obesity and the standpoint that obesity is a chronic disease that requires long term health care [61, 62].

Not all children and adolescents with severe obesity should undergo inpatient treatment, but also the ones that can profit sufficiently from outpatient care probably need ongoing support from health care professionals, their parents and the environment to regulate their food intake in such a way that it matches energy expenditure.

## Conclusions

In conclusion, the current study shows that the ability to self-regulate after an intensive, partly inpatient, multidisciplinary one year intervention for severe obesity in children and adolescents was not associated with the ability to maintain the achieved weight loss during the following year. Other factors that may modify this association in subgroups were not detected, apart from the control over eating by parents at the end of treatment that supports weight loss maintenance in participants with a low inhibitory control at the end of treatment. Factors that do explain the large range of long term outcomes still need to be elucidated.

## Abbreviations

AV: adjusted value
BMI: Body Mass Index
SD: standard deviation
SDS-BMI: Standard Deviation Score of Body Mass Index
SSRT: Stop Signal Reaction Time
